# Prevalence and Clinical Characteristics of Subclavian Steal Phenomenon/Syndrome in Patients with Acute Ischemic Stroke

**DOI:** 10.3390/jcm10225237

**Published:** 2021-11-10

**Authors:** Zoltan Bajko, Anca Motataianu, Adina Stoian, Laura Barcutean, Sebastian Andone, Smaranda Maier, Iulia-Adela Drăghici, Andrada Cioban, Rodica Balasa

**Affiliations:** 1 Department of Neurology, University of Medicine, Pharmacy, Sciences and Technology, 540136 Targu Mures, Romania; zoltan.bajko@umfst.ro (Z.B.); anca.motataianu@umfst.ro (A.M.); laura.barcutean@umfst.ro (L.B.); smaranda.maier@umfst.ro (S.M.); rodica.balasa@umfst.ro (R.B.); 2 First Neurology Clinic, Mures County Clinical Emergency Hospital, 540136 Targu Mures, Romania; adina.stoian@umfst.ro (A.S.); iuliab93@yahoo.com (I.-A.D.); andrada_farcas@yahoo.com (A.C.); 3 Department of Pathophysiology, University of Medicine, Pharmacy, Sciences and Technology, 540136 Targu Mures, Romania

**Keywords:** subclavian steal syndrome, subclavian steal phenomenon, ischaemic stroke

## Abstract

There are no published clinical studies regarding the prevalence of subclavian steal among acute ischemic stroke patients. The aim of this study was to evaluate the prevalence and clinical significance of subclavian steal among a large number of consecutive ischemic stroke patients. Materials and methods: We reviewed the medical records of 2192 consecutive cases of acute ischemic stroke at a tertiary neurology clinic in Targu Mures, Romania, between 2018 and 2020. In total, 47 patients (2.2%) were diagnosed with subclavian steal phenomenon/syndrome. Results: Stroke patients with associated steal phenomenon were significantly younger (64.2 ± 11.1 versus 70.2 ± 12.8, *p* = 0.005) and predominantly male (68.1%). From among the 47 patients with subclavian steal phenomenon, nine (19.1%) presented stroke symptomatology in the vertebrobasilar territory. Overall, 83.3% of the stroke patients with associated steal phenomenon presented cerebral infarction and 16.7% presented TIA. There was no difference between groups regarding the affected vascular territory (VB versus carotid). Large artery atherosclerosis was more frequent in the stroke group with associated steal phenomenon (81.3% versus 43.5%, *p* = 0.0033). The NIHSS score at admission was higher in the patient group with associated steal phenomenon, but there was no difference in mRS at discharge. Associated carotid artery occlusion was more frequent in the stroke group with steal phenomenon (*p* < 0.01). Smoking and peripheral arteriopathy were more frequent in the patient group with associated steal phenomenon. Of the nine symptomatic patients, five underwent revascularization treatment. Conclusions: The prevalence of subclavian steal phenomenon among acute ischemic stroke patients was not higher than in other cohorts with heterogenous peripheral vascular pathologies. Similar to the general population, in acute ischemic stroke patients, the associated subclavian steal behaved like a benign hemodynamical condition, without severe consequences.

## 1. Introduction

The vascular steal phenomenon occurs when the distal part of an occluded or severely stenosed artery is perfused via diverted blood flow from the bordering arterial territories. This situation may lead to ischemic symptomatology in the donor territory. The most common vascular steal effect is the subclavian one, which is known as subclavian steal syndrome (SSS) in symptomatic cases and subclavian steal phenomenon in asymptomatic cases, respectively [1,2,3,4,5,6].

The subclavian steal phenomenon is usually diagnosed incidentally during a routine cervical duplex ultrasound examination or during a routine clinical examination when a significant blood pressure difference between the upper extremities (>20 mmHg), a diminished or absent radial artery pulse, or a supraclavicular bruit is registered that prompts the examiner to ask for further investigations [7].

The duplex ultrasound examination can easily demonstrate reversed VA flow and significant subclavian artery stenosis; however, it is limited in the evaluation of VA origin because of its intrathoracic location. A latent or intermittent steal phenomenon can be evidenced with the aid of the reactive hyperemia test [8]. In symptomatic cases, especially where the need for interventional treatment arises, angiographic (computed tomography angiography (CTA), magnetic resonance angiography (MRA), and digital subtraction angiography (DSA)) confirmation is important [8,9,10,11,12,13].

Subclavian steal is considered a harmless hemodynamic phenomenon and a marker of atherosclerotic disease; however, in symptomatic cases, revascularization interventions are sometimes necessary, especially when there are refractory symptoms, such as repetitive vertebro-basilary transient ischemic attacks (TIAs) or symptomatic arm ischemia [14]. During more than two years of follow-up of a large group of SSS patients, several stroke complications were reported (in 26% of cases), but in most of the cases, the anterior circulation was affected. The only significant risk factor for stroke in patients with SSS was the presence of symptoms on presentation [15].

There are limited epidemiological data in the literature regarding the prevalence of SSS. Most of the data have come from ultrasound laboratories, where heterogenous patient groups have been examined with different cardiovascular and cerebrovascular pathologies [14,16,17].

To our knowledge, there are no published clinical studies regarding the prevalence of subclavian steal among acute ischemic stroke patients. The aim of this study was to evaluate the prevalence and clinical significance of SSS among a large number of consecutive ischemic stroke patients.

## 2. Materials and Methods

We reviewed the medical records of 2192 consecutive cases of acute ischemic stroke at a tertiary neurology clinic in Targu Mures, Romania, between 2018 and 2020. The ischemic stroke cases were selected from our stroke database based on the discharge diagnosis. The main inclusion criteria were a diagnosis of acute cerebral infarction or TIA and an available complete duplex ultrasound examination report on the cervical vessels. Overall, 55 patients were excluded owing to incomplete duplex ultrasound data. Data from 2137 patients were analyzed.

We documented the detailed risk-factor profile, demographic data, and main ischemic stroke characteristics, such as stroke type, affected vascular territory, Trial of ORG 10,172 in Acute Stroke Treatment (TOAST) [18] classification, and stroke severity. Severity of stroke was measured using the National Institute of Health Stroke Scale (NIHSS) [19] at admission and the modified Rankin scale (mRS) [20] at discharge.

Ethical approval was obtained from the institutional review board of the hospital (approval number: 33881).

The diagnosis of subclavian steal was based on the following criteria: retrograde VA blood flow, evidence of significant subclavian or brachiocephalic artery stenosis or occlusion, and patency of the vertebral arteries and the basilar artery. Additional vascular imaging was performed in 11 cases (CTA in 6 and DSA in 5 cases), which revealed severe brachiocephalic artery stenosis in one case, subclavian occlusion in 6 cases, and severe stenosis in 5 cases.

The steal phenomenon was considered incomplete in cases with the following VA Doppler waveforms: systolic deceleration (decrease in systolic flow velocity and unchanged diastolic flow) or alternating flow (retrograde flow during systole and anterograde flow during diastole). The steal phenomenon was considered complete in cases of complete reversal of flow at rest during the entire cardiac cycle.

The steal phenomenon was considered symptomatic in cases of TIAs or cerebral infarction in the vertebrobasilar territory. The reactive hyperemia test was performed and was positive in all cases with a definite diagnosis of the subclavian steal phenomenon, revealing an enhanced reverse component of the vertebral artery blood flow.

Statistical analysis was carried out using descriptive methods, continuous variables were expressed with their means and standard deviations (SDs), or frequencies and percentages. Statistical hypotheses were tested using standard statistics, and evaluated for significance using two-sample t-tests or the Mann–Whitney U test. Categorical variables were analyzed using Fisher’s exact test. Separate comparisons were performed for patients with and without the subclavian steal phenomenon, and for the symptomatic and asymptomatic subclavian steal groups.

## 3. Results

### 3.1. Demographic Data

Overall, 2.2% of the acute stroke patients were diagnosed with the subclavian steal phenomenon. The stroke patients with the associated steal phenomenon were significantly younger (64.2 ± 11.1 versus 70.2 ± 12.8, *p* = 0.005) and predominantly male (68.1%) (Table 1).

### 3.2. Stroke Characteristics

From among the 47 patients with the subclavian steal phenomenon, nine (19.1%) presented stroke symptomatology in the vertebrobasilar territory. Overall, 83.3% of the stroke patients with the associated steal phenomenon presented cerebral infarction and 16.7% presented TIA. The percentage of TIA was higher in this group compared with the stroke group without the steal phenomenon. There was no difference between groups regarding the affected vascular territory (VB versus carotid).

Regarding the TOAST classification, large artery atherosclerosis was more frequent in the stroke group with the associated steal phenomenon (81.3% versus 43.5%, *p* = 0.0033). The other unknown group was more frequent in the patient group without the steal phenomenon.

The NIHSS score at admission was higher in the patient group with the associated steal phenomenon, but there was no difference in mRS at discharge.

Associated carotid artery occlusion was more frequent in the stroke group with the steal phenomenon (*p* < 0.01) (Table 1).

### 3.3. Risk Factor Profile

Smoking and peripheral arteriopathy were more frequent in the patient group with the associated steal phenomenon, and atrial fibrillation was more frequent in the stroke group without the steal phenomenon. There were no other significant differences between groups (Table 1).

### 3.4. Symptomatic and Asymptomatic Steal Groups

The mean age of the patients in the symptomatic group was significantly lower (*p* = 0.002). TIA was more frequent in the symptomatic group. There was no difference between groups regarding the TOAST classification. The steal phenomenon was more frequently incomplete in both groups. The left-sided affection was predominant in both groups. There was no difference regarding the risk-factor profile. In patients with cerebral infarction, NIHSS at admission and mRS at discharge were significantly lower in the symptomatic group. Regarding the risk-factor profile, the triglyceride level was significantly higher in the symptomatic group. There was no difference regarding associated carotid atherosclerosis. (Table 2).

Severe subclavian stenosis and occlusion on angiography was significantly associated with the complete steal phenomenon on duplex ultrasound (*p* = 0.04).

**Figure 1:**
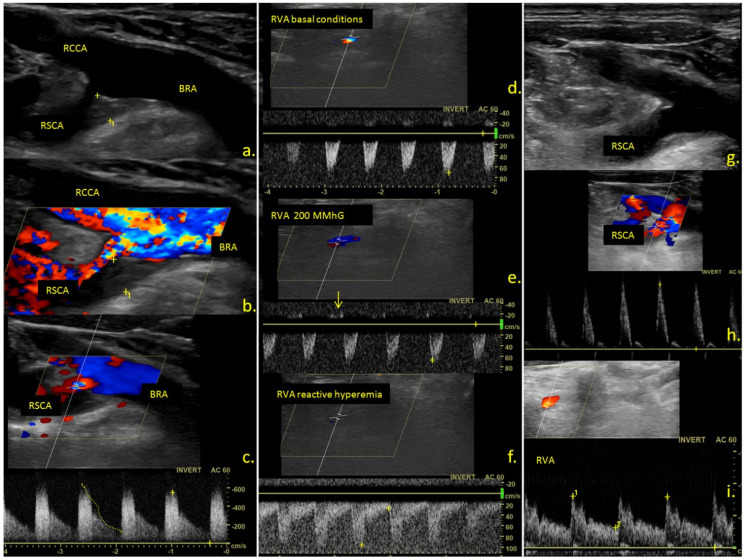
Ultrasound examination of the cervical vessels. (**a**) B-mode; (**b**) color mode; (**c**) triplex mode ultrasound examination revealing a hypoechoic atherosclerotic plaque at the origin of the right subclavian artery leading to severe stenosis. (**d**–**f**) triplex mode examination of the right vertebral artery in basal conditions (**d**) during compression of the right arm (**e**) and reactive hyperemia (**f**) revealing negative midsysolic and no diastolic flow in basal conditions (**d**); the appearance of a minimal positive dyastolic flow during the compression of the arm ((**e**), arrow) and complete flow inversion during the entire cardiac cycle during reactive hyperemia of the arm (**f**); (**g**–**i**) ultrasound examination after balloon angioplasty of the right subclavian artery, revealing a significant reduction of the grade of stenosis. ((**g**) B-mode image, (**h**) triplex mode image, showing a normal triphasic subclavian waveform), and a normal, anterograd flow in the right vertebral artery (**i**). RCCA—right common carotid artery, RSCA—right subclavian artery, BRA—brachiocephalic artery, RVA—right vertebral artery.

Of the nine symptomatic patients, five underwent revascularization treatment (three with percutaneous transluminal angioplasty and stenting and two with subclavian-carotid by-pass). The rest of the patients were treated conservatively with antiplatelets, statins, and risk-factor control, because of isolated transient symptoms, mild symptomatology, or the patient’s preference. Figure 1 presents the duplex ultrasound examination of a 43-year-old male patient with repeated transient ischemic attacks in the vertebro-basilar territory secondary to a severe right sided subclavian artery stenosis, resulting in the subclavian steal phenomenon, who underwent balloon angioplasty, with significant reduction of the subclavian stenosis and disappearance of the steal phenomenon.

## 4. Discussion

This study is, to the best of our knowledge, the first to investigate the prevalence and clinical characteristics of the subclavian steal phenomenon among a large number of consecutive acute ischemic stroke patients. The prevalence of 2.2% in the stroke patients was within the range of the previously reported results from heterogeneous patient groups with different vascular pathologies (0.6–6.4%) [16,21,22,23,24]. Tan et al. reported a prevalence of 2.7% among 1860 patients who underwent duplex ultrasound examination of the extracranial brain-supplying arteries in a neuro-ultrasound laboratory [16]. Labropoulus et al. analyzed data from 7881 patients who underwent duplex ultrasound examination over a 6-year period. They observed a prevalence of 5.4%, and most of the lesions appeared in the left side. Only 7.4% of the patients were symptomatic [17], which was significantly lower compared with the current cohort in which 19.1% of the patients were symptomatic, despite the fact that we defined this group as including only those with ischemic cerebrovascular symptoms.

The majority of the stroke patients with the associated subclavian steal phenomenon presented carotid territory symptoms. This observation was similar to those published by several authors previously [21,25,26], and can be explained by the concomitant widespread affection of the carotid circulation in this population. This was also observed in the current cohort, in which patients with the subclavian steal phenomenon presented carotid artery occlusion or severe stenosis more frequently than the control group.

Notably, the stroke patients with the associated subclavian steal phenomenon in the current cohort were significantly younger. The age difference was also evident between the symptomatic and asymptomatic subclavian steal groups, with the former being significantly younger. A possible explanation for this finding was the accelerated atherosclerosis and insufficient collateral circulation in the younger patients.

Regarding the risk-factor profile, in the current cohort, smoking and peripheral arteriopathy were significantly more frequent in patients with the associated subclavian steal phenomenon. There were no significant differences between symptomatic and asymptomatic groups. Similar results were reported in the literature regarding the association of smoking with subclavian artery stenosis. Shadman et al. analyzed the prevalence and risk-factor profile of two free-living and two clinical populations. Subclavian artery stenosis was significantly associated with past and current smoking and higher levels of systolic blood pressure [27].

The main limitations of the current study were its retrospective design and that the blood pressure differences between the right and left arm were not documented in the database, and so could not be included in the statistical analysis.

In the current cohort, the associated subclavian steal phenomenon did not negatively influence the gravity and short-term outcome of ischemic stroke patients. Even the symptomatic patients presented only TIA or minor stroke with excellent short-term outcomes.

## 5. Conclusions

The prevalence of the subclavian steal phenomenon among acute ischemic stroke patients was not higher than in other cohorts with heterogenous peripheral vascular pathologies. Similar to the general population, in acute ischemic stroke patients, the associated subclavian steal behaved like a benign hemodynamical condition, without severe consequences. Interventional revascularization was warranted only in symptomatic cases with repetitive symptomatology.

## Figures and Tables

**Table 1:** The main demographical and clinical characteristics of the stroke groups with and without the associated subclavian steal phenomenon.

			Subclavian Steal Group *n* = 47	Without Subclavian Steal *n* = 2090	*p* Value
Mean age			64.2 ± 11.1 years	70.2 ± 12.8 years	0.0005
Sex			32M (68.1%) 15F (31.9)	1070 (51.2) 1020 (48.8)	0.19
Stroke Type	TIA		8 (17.0)	177 (8.46)	0.08
	Cerebral infarction		39 (83.0)	1913 (91.5)	0.74
Stroke territory	Carotid		37 (78.7)	1598 (76.5)	0.91
	Left		18 (38.3)	943 (45.1)	0.59
	Right		19 (40.4)	655 (31.3)	0.31
	VB		10 (21.3)	492 (23.5)	0.86
TOAST	Large artery atherosclerosis		38 (80.6)	909 (43.5)	0.0033
	Small vessel disease		3 (6.4)	364 (17.4)	0.11
	Cardioembolism		5 (10.6)	458 (21.9)	0.14
	Other, known		1 (2.1)	69 (3.3)	0.4
	Other unknown		0	197 (9.4)	0.03
Smoking			23 (48.9)	496 (23.7)	0.0092
Alcool			7 (14.9)	251 (12.0)	0.65
Fibrillation			5 (10.6)	524 (25.1)	0.05
Arteriopathy			11 (23.4)	180 (8.6)	0.0071
Hypertension			41 (87.2)	1932 (92.4)	0.74
Diabetes			10 (21.3)	502 (24.0)	0.86
COPD			5 (10.6)	210 (10.0)	0.81
Renal failure			3 (6.4)	130 (6.2)	0.99
NIHSS admission			7.3 ± 5.1	5.4 ± 6.2	*0.02*
mRS at discharge			2.02 ± 1.7	2.2 ± 1.8	0.28
Cholesterol			190.9 ±45.1	183.6 ±49.8	0.30
Triglyceride			153.2 ± 71.3	143.8 ± 122.4	0.46
Glucose			120.9 ± 46.6	129.3 ± 56.4	0.22
Associated carotid atherosclerosis	Left	occlusion	6 (12.8)	45 (2.2)	0.0013
		>70%	3 (6.4)	37 (1.8)	0.06
		50–70%	4 (8.5)	119 (5.7)	0.52
		<50%	22 (46.8)	600 (28.7)	0.08
	Right	occlusion	8 (17.0)	35 (1.7)	<0.001
		>70%	1 (2.1)	35 (1.7)	0.56
		50–70%	2 (4.3)	105 (5.0)	0.99
		<50%	24 (51.1)	588 (28.1)	0.03
Controlateral vertebral		occlusion	2 (4.3)	18 (0.8)	0.07
		stenosis	0	43 (2.1)	0.99

**Table 2:** The main demographical and clinical characteristics of the patient groups with associated symptomatic and asymptomatic subclavian steal.

			Symptomatic Group (*n* = 9)	Asymptomatic Group (*n* = 38)	*p* Value
Mean age			52.8	66.9	0.002
Sex (M/F)			7/2	25/13	0.78
Stroke Type	TIA		5	3	0.019
	Ischemic stroke		4	35	0.36
Stroke territory	Carotid		0	38	0.0037
	Left		0	18	0.053
	Right		0	20	0.047
TOAST	Large artery atherosclerosis		8	29	0.79
	Small vessel disease		0	3	1.0
	Cardioembolism		0	5	0.57
	Other, known		1	0	0.2
	Other unknown		0	1	1.0
Steal type	Incomplete		6	26	1.0
	Complete		3	12	1.0
Steal side	Right		3	25	0.51
	Left		5	13	0.5
	Bilateral		1	0	0.2
Smoking			3	20	0.73
Alcool			0	7	0.58
Fibrillation			0	5	0.57
Arteriopathy			1	6	1.0
Hypertension			4	36	0.36
Diabetes			3	7	0.42
COPD			0	5	0.57
Renal failure			0	3	1.0
NIHSS admission			0.5 ± 1.0	8.2 ± 4.7	<0.001
mRS at discharge			0.4 ± 0.9	2.5 ± 1.6	<0.0001
Cholesterol			183.9 ± 50.6	192.6 ± 44.3	0.66
Triglyceride			210.1 ± 77.3	137.8 ± 62.7	0.05
Glucose			134.6 ± 62.2	117.5 ± 43.2	0.44
Associated carotid atherosclerosis	Left	occlusion	0	6	0.57
		>70%	1	2	0.49
		50–70%	1	3	1.0
		<50%	4	18	1.0
	Right	occlusion	0	8	0.32
		>70%	0	1	1.0
		50–70%	0	2	1.0
		<50%	3	20	0.73
Controlateral vertebral		occlusion	1	1	0.36
		stenosis	0	0	1.0

## Data Availability

Data is available from the corresponding author on reasonable request.

## References

[1] Toole J.F., McGraw C.P. (1975). The steal syndromes. Annu. Rev. Med..

[2] Contorni L. (1960). The vertebro-vertebral collateral circulation in obliteration of the subclavian artery at its origin. Minerva Chir..

[3] Reivich M., Holling H.E., Roberts B., Toole J.F. (1961). Reversal of blood flow through the vertebral artery and its effect on cerebral circulation. N. Engl. J. Med..

[4] Barnett H.J., Wortzman G., Gladstone R.M., Lougheed W.M. (1970). Diversion and reversal of cerebral blood flow. External carotid artery “steal”. Neurology.

[5] Maier S., Bajkó Z., Moțățăianu A., Maier A., Raicea V., Bardas A., Bălașa R. (2014). Subclavian double steal syndrome presenting with cognitive impairment and dizziness. Rom. J. Neurol..

[6] Cala L.A., Armstrong B.K. (1972). A “triple-steal syndrome” resulting from innominate and left subclavian arterial occlusion. Aust. N. Z. J. Med..

[7] Alcocer F., David M., Goodman R., Jain S.K., David S. (2013). A forgotten vascular disease with important clinical implications. Subclavian steal syndrome. Am. J. Case Rep..

[8] Päivänsalo M., Heikkilä O., Tikkakoski T., Leinonen S., Merikanto J., Suramo I. (1998). Duplex ultrasound in the subclavian steal syndrome. Acta Radiol..

[9] Konda S., Dayawansa S., Singel S., Huang J.H. (2015). Pseudo subclavian steal syndrome: Case report. Int. J. Surg. Case Rep..

[10] Kargiotis O., Siahos S., Safouris A., Feleskouras A., Magoufis G., Tsivgoulis G. (2016). Subclavian Steal Syndrome with or without Arterial Stenosis: A Review. J. Neuroimaging.

[11] Bajkó Z., Bălaşa R., Moţăţăianu A., Bărcuţean L., Stoian A., Stirbu N., Maier S. (2016). Malignant middle cerebral artery infarction secondary to traumatic bilateral internal carotid artery dissection. A case report. J. Crit. Care Med..

[12] Filep R.C., Bajko Z., Simu I.P., Stoian A. (2020). Pseudo dissection of the internal carotid artery in acute ischemic stroke. Acta Neurol. Belg..

[13] Bajkó Z., Maier S., Moţăţăianu A., Bălaşa R., Vasiu S., Stoian A., Andone S. (2018). Stroke secondary to traumatic carotid artery injury A case report. J. Crit. Care Med..

[14] Osiro S., Zurada A., Gielecki J., Shoja M.M., Tubbs R.S., Loukas M. (2012). A review of subclavian steal syndrome with clinical correlation. Med. Sci. Monit..

[15] Tan X., Bai H.X., Wang Z., Yang L. (2017). Risk of stroke in imaging-proven subclavian steal syndrome. J. Clin. Neurosci..

[16] Tan T.Y., Schminke U., Lien L.M., Tegeler C.H. (2002). Subclavian steal syndrome: Can the blood pressure difference between arms predict the severity of steal?. J. Neuroimag..

[17] Labropoulos N., Nandivada P., Bekelis K. (2010). Prevalence and impact of the subclavian steal syndrome. Ann. Surg..

[18] Adams H.P., Bendixen B.H., Kappelle L.J., Biller J., Love B.B., Gordon D.L., Marsh E.E. (1993). Classification of subtype of acute ischemic stroke. Definitions for use in a multicenter clinical trial. TOAST. Trial of Org 10172 in Acute Stroke Treatment. Stroke.

[19] Kasner S.E. (2006). Clinical interpretation and use of stroke scales. Lancet Neurol..

[20] Quinn T.J., Dawson J., Walters M.R., Lees K.R. (2009). Reliability of the modified Rankin Scale: A systematic review. Stroke.

[21] Hennerici M., Klemm C., Rautenberg W. (1988). The subclavian steal phenomenon: A common vascular disorder with rare neurologic deficits. Neurology.

[22] Fields W.S., Lemak N.A. (1972). Joint Study of extracranial arterial occlusion. VII. Subclavian steal—A review of 168 cases. JAMA.

[23] Lord R.S., Adar R., Stein R.L. (1969). Contribution of the circle of Willis to the subclavian steal syndrome. Circulation.

[24] Bornstein N.M., Norris J.W. (1986). Subclavian steal: A harmless haemodynamic phenomenon?. Lancet.

[25] Smith J.M., Koury H.I., Hafner C.D., Welling R.E. (1994). Subclavian steal syndrome. A review of 59 consecutive cases. J. Cardiovasc. Surg..

[26] Nicholls S.C., Koutlas T.C., Strandness D.E. (1991). Clinical significance of retrograde flow in the vertebral artery. Ann. Vasc. Surg..

[27] Shadman R., Criqui M.H., Bundens W.P., Fronek A., Denenberg J.O., Gamst A.C., McDermott M.M. (2004). Subclavian artery stenosis: Prevalence, risk factors, and association with cardiovascular diseases. J. Am. Coll. Cardiol..

